# The Life Mission Theory VI. A Theory for the Human Character: Healing with Holistic Medicine Through Recovery of Character and Purpose of Life

**DOI:** 10.1100/tsw.2004.142

**Published:** 2004-09-24

**Authors:** Søren Ventegodt, Maximilian Kromann, Niels Jørgen Andersen, Joav Merrick

**Affiliations:** ^1^ The Quality of Life Research Center, Teglgårdstræde 4-8, DK-1452 Copenhagen K, Denmark; ^2^ The Research Clinic for Holistic Medicine, Copenhagen, Denmark; ^3^ Norwegian School of Management, Sandvika, Norway; ^4^ National Institute of Child Health and Human Development, Office of the Medical Director, Division for Mental Retardation, Ministry of Social Affairs, Jerusalem and Zusman Child Development Center, Division of Pediatrics and Community Health, Ben Gurion University, Beer-Sheva, Israel

**Keywords:** quality of life, QOL, philosophy, human development, holistic medicine, alternative medicine, public health, life mission theory, public health, personal development, purpose of life, character, spiritual health, hormonal balance, Denmark, Israel, Norway

## Abstract

The human character can be understood as an extension of the life mission or purpose of life, and explained as the primary tool of a person to impact others and express the purpose of life. Repression of the human character makes it impossible for a person to realize his personal mission in life and, therefore, is one of the primary causes of self-repression resulting in poor quality of life, health, and ability. From Hippocrates to Hahnemann, repression of physical, mental, and spiritual character can be seen as the prime cause of disease, while recovery of character has been the primary intention of the treatment. In this paper, human character is explained as an intersubjective aspect of consciousness with the ability to influence the consciousness of another person directly. To understand consciousness, we reintroduce the seven-ray theory of consciousness explaining consciousness in accordance with a fractal ontology with a bifurcation number of seven (the numbers four to ten work almost as well). A case report on a female, aged 35 years, with severe hormonal disturbances, diagnosed with extremely early menopause, is presented and treated according to the theory of holistic existential healing (the holistic process theory of healing). After recovery of her character and purpose of life, her quality of life dramatically improved and hormonal status normalized. We believe that the recovery of human character and purpose of life was the central intention of Hippocrates and thus the original essence of western medicine. Interestingly, there are strong parallels to the peyote medicine of the Native Americans, the African Sangomas, the Australian Aboriginal healers, and the old Nordic medicine. The recovery of human character was also the intention of Hahnemann's homeopathy. We believe that we are at the core of consciousness-based medicine, as recovery of purpose of life and human character has been practiced as medicine in most human cultures throughout time. We believe that such recovery can help some (motivated) patients to survive, even with severe disease.

## INTRODUCTION

In our experience, every human being has a distinct character, but the human character is a deep mystery that is not connected to anatomy or biochemistry, but directly to consciousness and personal style of behavior. It is often presumed that consciousness is private, but our theory of human character states the opposite and we believe that character is an aspect of consciousness, which is interpersonal and open to the experience of another person.

The reason for making such a "weird" theory is that we have often experienced that patients have repressed their character and only when this repression is reversed can their purpose of life be known and realized. This opens up the interesting perspective that our human character serves our purpose of life directly through working on the other and that repression of our character early in life to fit into our family causes severe, but reversible, damage to our soul and wholeness.

In the clinic, working with the holistic process of healing, we often find that the recovery of character is connected to improved quality of life, physical, and mental health. We assume that this improvement can be explained by the life mission theory[1,2,3,4,5,6]. Interestingly, many early physicians from Hippocrates (460–377 BCE) to Samuel Hahnemann (1755–1843, the father of homeopathy) also thought that the personality, or character of "geist" [spirit] and "gemut" [body-mind], as Hahnemann worded it, was a prime cause of disease[7].

The human character is an issue that takes many words to describe (Table 1) and it has been one of the most important concepts in the human arts, from the epic and prose writings of all time to any kind of performance, from the theater to the martial arts. Let us give a famous example from a writer (Fyodor (Mikhaylovich) Dostoevsky (1821–1881)[8]) who mastered the description of human character:

> "One of them was of medium height, about twenty – seven years old, with curly, almost black hair, and small fiery grey eyes. He had a broad, flat nose and high cheekbones; his thin lips were constantly twisting into a sort of impudent, mocking and even malicious smile; but his forehead was high and well formed and made up for the lack nobility in the lower part of his face. Especially notable was the deathly pallor of his face, which gave the young man's whole physiognomy an exhausted look, despite his rather robust build, and at the same time suggested something passionate, to the point of suffering, which was out of harmony with his insolent and coarse smile and his sharp, self-satisfied face."

### Character in Existential Philosophy

Philosophy has spent a lot of time on the subject of the human character. Let us just recontemplate a piece of the well-known work by Jean-Paul Sartre (1905–1980) on the dramatic loss of the physical character that follows giving up personal freedom, a concept of resignation very close to self-denial, which we see as the essential cause of the loss of quality of life, health, and ability in the life mission theory[2]:

**TABLE 1**
**Some English Words* Used to Describe Human Character**

acquisitive, adaptable, admirable, adorable, adorable, aggressive, aiming, airy, amazing, ambivalent, analytic, angry, anxious, anxious, apathetic, assertive, athletic, attractive, authentic, avaricious, awful, balanced, barbarian, baroque, bitter, bitter, bizarre, blissful, bold, boyish, brainy, brave, brotherly, burdensome, calculative, calm, capable, caring, caring, cautious, celebrative, cheerful, clever, committed, compassion, compassionate, competitive, concerned, contemplating, controlling, cool, coping, corny, courageous, crazy, critical, crude, curious, dealer, decadent, deceptive, decisive, delicate, delicious, depending, depraved, depressive, deranged, desirable, desolate, despiteful, destructive, detached, different, direct, disciplined, disengaged, disgusting, distinctive, dominating, dreadful, dreamful, dreamy, dubious, dutiful, effective, eloquent, emotional, empathy, energetic, engaged, enthusiastic, envious, erotic, evil, exotic, extreme, forgiving, fragile, fragmented, frantic, free-spirited, frenzy, friendly, futile, glorifying, gluttonous, grandiose, grounded, heedless, helpful, helpless, heroic, high, honest, horny, horrifying, humorous, hyperactive, hysterical, indulge, innovative, insightful, inspiring, intense, intimidating, introspective, intrusive, intuitive, involved, irrational, isolated, jovial, joyful, kind, kinky, labile, lame, lazy, leading, liar, likable, loving, loyal, lustful, magnanimous, malicious, manipulative, megalomaniac, melancholy, misogyny, moody, mysterious, nihilistic, non-human, numb, obsessive, obstructive, opportunistic, oppressive, optimistic, opulent, outstanding, painful, panicle, paralyzed, paranoid, patient, peaceful, perceptive, perfectionist, pessimistic, philanthropic, philosophical, plain, pleasant, popular, possessive, potent, powerful, pragmatic, pretentious, pride, prideful, private, productive, profound, prostitute, proud, provocative, prudent, psychic, psycho, psychotic, quiet, raging, reactive, realistic, reassured, redemptive, reflexive, relaxed, relentless, reliable, resentful, resilient, resourceful, responsive, revelatory, robotic, , robust, ruthless, sad, scary, scattered, schizoid, scornful, secretive, seductive, self deceptive, self neglecting, self-assured, self-contained, selfish, selfless, self-possessed, self-promoting, self-reliant, sensible, sensitive, serenity, shameful, sharing, sheer, shining, silent, simple, sinful, sisterly, skilful, sloppy, slothful, smart, solitary, solitude, soulful, special, speculative, spiritual, spontaneous, spooky, stable, steady, steady, stoical, strange, strong, stubborn, stupid, sullen, supportive, suspicious, sweet, sympathetic, tempting, tenacious, tender, tentative, thoughtful, trippy, trustful, trustworthy, trusty, twisted, unconscious, unique, unusual, valiant, vengeant, violent, virtues, visionary, visionary, vulgar, warm, weird, wicked, willful, wise, wishful, worried

* Some words are normally considered positive, others are considered to be negative qualities. Interestingly, all of them can be seen as genuine aspects of human character and they are thus not evil, but mere tools for the soul. The intentions of the soul determine whether a dangerous or potentially negative streak is having positive or negative consequences. Often a negative quality will turn out to be a suppressed positive quality of the person's character, and in the same way, a positive character can turn out to be false and created by repression of a true "negative" character, serving the soul in its mission.

> "Take the example of a woman who has consented to go out with a particular man for the first time. She knows very well the intentions, which the man who is speaking to her cherishes regarding her. She knows also that it will be necessary sooner or later for her to make a decision. But she does not want to realize the urgency; she concerns herself only with what is respectful and discreet in the attitude of her companion. She does not apprehend this conduct as an attempt to achieve what we call "the first approach"; that is, she does not want to see the possibilities of temporal development, which his conduct presents. She restricts this behaviour to what is in the present; she does not wish to read in the phrases, which he addresses, to her anything other than their explicit meaning. If he says to her, "I find you so attractive!" she disarms this phrase of its sexual background; she attaches to the conversation and to the behaviour of the speaker, the immediate meanings, which she imagines as objective qualities. The man who is speaking to her appears to her sincere and respectful as the table is round or square, as the wall colouring is blue or grey. The qualities thus attached to the person she is listening to are in this way fixed in a permanence like that of things, which is no other than the projection of the strict present of the qualities into the temporal flux.
>
> This is because she does not quite know what she wants. She is profoundly aware of the desire, which she inspires, but the desire cruel and naked would humiliate and horrify her. In order to satisfy her, there must be a feeling, which is addressed wholly to her *personality*- that is, to her full freedom- and which would be recognition of her freedom.

> But at the same time this feeling must be wholly desire; that is, it must address itself to her body as object. This time then she refuses to apprehend the desire for what it is; she does not even give it a name; she recognizes it only to the extent that it transcends itself toward admiration, esteem, respect and that it is wholly absorbed in the more refined forms which it produces, to the extent of no longer figuring anymore as a sort of warmth and density. But then suppose he takes her hand. This act of her companion risks changing the situation by calling for an immediate decision. To leave the hand there is to consent in herself to flirt, to engage herself. To withdraw it is to break the troubled and unstable harmony, which gives the hour its charm. The aim is to postpone the moment of decision as long as possible. We know what happens next; the young woman leaves her hand there, but she *does not notice* that she is leaving it. She does not notice because it happens by chance that she is at this moment all intellect. She draws her companion up to the most lofty regions of sentimental speculation; she speaks of life, of her life, she shows herself in her essential aspect – a personality, a consciousness. And during this time the divorce of the body from the soul is accomplished; the hand rests inert between the warm hands of her companion- neither consenting nor resisting – a thing."(Jean-Paul Sartre[9])

It is very interesting that physical and sexual character are so closely related, that giving up one means giving up the other also, which seems to be a crucial point of Sartre in this text. The problems of being yourself are as old as mankind itself. Losing contact with the real self through repression of physical-sexual character has been a classical existential theme. Let us take one more example, where Sartre[10] illustrates the quality of life, lack of consciousness, and direction in life, after the repression of the human character is completed. Interestingly many patients live in such a diffuse state of being:

> "There is a white hole in the wall, a mirror. It is a trap. I know I am going to let myself be caught in it. I have. The grey thing appears in the mirror. I go over and look at it, I can no longer get away.
>
> It is the reflection of my face. Often in these lost days I study it. I can understand nothing of this face. The faces of others have some sense, some direction. Not mine. I cannot even decide whether it is handsome or ugly. I think it is ugly because I have been told so. But it doesn't strike me. At heart, I am not even shocked that anyone can attribute qualities of this kind to it, as if you called a clod of earth or a block of stone beautiful of ugly…………………My glance slowly and wearily travels over my forehead, my cheeks: it finds nothing firm, it is stranded. Obviously there are a nose, two eyes and a mouth, but none of it makes sense, there is not even a human expression. Yet Anny and Vélines thought I looked so alive: perhaps I am too used to my face. When I was little, my Aunt Bigeois told me "If you look at yourself too long in the mirror, you'll see a monkey." I must have looked at myself longer than that: what I see is well below the monkey, on the fringe of the vegetable world, at the level of jellyfish. It is alive, I can't say it is isn't; but this was not the life that Anny contemplated: I see a slight tremor, I see the insipid flesh blossoming and palpitating abandon. The eyes especially are horrible seen so close. They are glassy, soft, blind, redrimmed, they look like fish scales. I lean all my weight on the porcelain ledge, I draw my face closer until it touches the mirror. The eyes, nose and mouth disappear: nothing human is left. Brown wrinkles show on each side of the feverish swelled lips, crevices, mole holes. A silky white down covers the great slopes of the cheeks, two hairs protrude from the nostrils: it is a geological embossed map. And, in spite of everything, this lunar world is familiar to me. I cannot say I *recognize* the details. But the whole thing gives me an impression of something seen before which stupefies me: I slip quietly off to sleep." (Jean-Paul Sartre[10])

## Character in Medicine: Understanding Hippocrates' Medicine, Hahnemann's Homeopathy, and Native American Medicine

The human character has been taken into serious consideration by old physicians in the tradition of Hippocrates[11] and Hahnemann[7]. The theory behind Hahnemann's controversial homeopathy can be interpreted as an attempt at recovery of the human character, if the character is seen as a prolongation of the "life force", the basic intention behind the individual, which we call the purpose of life in the life mission theory[2]. The Hippocratic writers have several theories of healing related to the human character, among which the difficult theory on humours related to the four elements (a "four ray" theory, please see below) is one of the most famous.

Looking at the *Organon of the Medical Art*[7] (by Hahnemann, his famous and most important book, where he established the art of homeopathy and defined both the physician, the nature, and objective of medicine), we see that he understood the real cause of disease as the depression of physical constitution (= physical character, see §5.1), and the psychic and spiritual character (§5.2) (please note that Hahnemann said the character of the "*Geist*" = spirit and the "*Gemüt*" = *psyche*, often wrongly translated into English as "the emotional and mental character"):

> §1
> The physician's highest and *only* calling is to make the sick healthy, to cure, as it is called.
>
> §2
> The highest ideal of cure is the rapid, gentle and permanent restoration of health; that is, the lifting and annihilation of the disease in its entire extent in the shortest, most reliable, and least disadvantageous way, according to clearly realizable (in-seeable) principles. (Compare to the square curve paradigm[12])
>
> §5
> It will help the physician to bring about a cure if he can find out the data of the most probable *occasion* of an acute disease, and the most significant factors in the entire history of a protracted wasting sickness, enabling him to find out its *fundamental cause*. The fundamental cause of a protracted wasting sickness mostly rests upon chronic miasm. In these investigations, the physician should take into account the patient's:
> 1. discernible body constitution (especially in cases of protracted disease) (Compare physical character),
> 2. [spiritual,] mental and emotional character (character of the *Geist* and the *Gemüt*),
> 3. occupations,
> 4. lifestyle and habits,
> 5. civic and domestic relationships (relationships outside and within the home),
> 6. age,
> 7. sexual function, etc.
>
> §9
> In the healthy human state, the spirit-like life force (autocracy) that enlivens the material organism as dynamis, governs without restriction and keeps all parts of the organism in admirable, harmonious, vital operation, as regards both feelings and functions, so that our indwelling, rational spirit can freely avail itself of this living, healthy instrument for the higher purposes of our existence.
>
> §10

> The material organism, thought of without life force, is capable of no sensibility, no activity, no self-preservation. It derives all sensibility and produces its life functions solely by means of the immaterial wesen (the life principle, the life force) that enlivens the material organism in health and in disease.
>
> §11
> When a person falls ill, it is initially only this spirit-like, autonomic life force (life principle), everywhere present in the organism, that is mistuned through the dynamic influence of a morbific agent inimical to life. Only the life principle, mistuned to such abnormality can impart to the organism the adverse sensations and induce in the organism the irregular functions that we call disease. The life principle is a power-wesen invisible in itself, only discernible by its effects on the organism. Therefore, its morbid mistunement only makes itself known (discernible) by manifestations of disease in feelings and functions (the only aspects of the organism accessible to the senses of the observer and medical-art practitioner). In other words, the morbid mistunement of the life principle makes itself discernible by disease symptoms; in no other way can it make itself known.

Fig. 1 shows the principle of the continuum of consciousness from the essence of the being, the purpose of life, to the energy of the physical body.

**ME**

Life purpose
Spiritual Character
Mental Character
Physical Character
VVVVV
**We are connecting**
ΛΛΛΛΛ
Physical Character
Mental Character
Spiritual Character
Life purpose

**YOU**

**FIGURE 1.** The purpose of life is manifested through the spiritual, mental, and physical character of the person and this character makes it possible for us to connect, communicate, and influence each other.

Fig. 2 is another version drawn (by computer) by a 35-year-old female patient of the connection between the purpose of life (the core) and the spiritual, mental, and physical character, the latter carrying the tools for impacting the other (harshly drawn by the patient who was still working on becoming more sophisticated in understanding her own character).

The physical character has been a main focus of theater plays, art, and philosophy[8,9]. The spiritual character has been the main focus of the great mystics like the Greek-Armenian mystic Georgii Ivanovitch Gurdjieff (1866–1949), who mapped nine fundamental spiritual characters in his enneagram model[13]. The mental character and all the different human intelligences, often seven, have been the issue of ancient research in human potential and are the focus of much of the new-age literature[14,15,16].

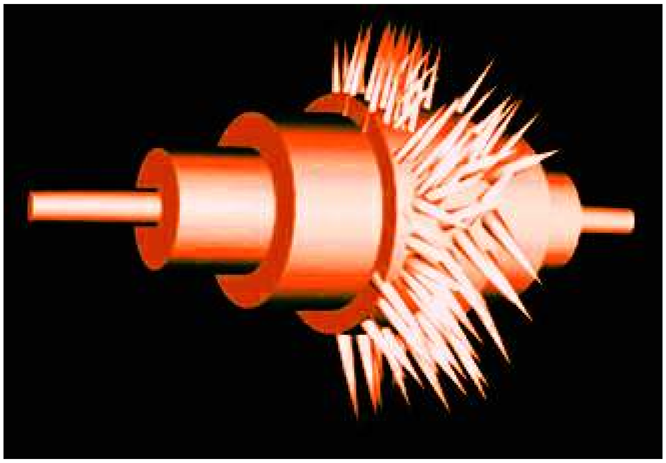

**FIGURE 2**. The human character is an extension of the life purpose. A 35-year-old female patient, architect, made this drawing using her favorite computer program. It shows in the center the purpose of life giving energy to the harsh and effective physical character through the spiritual and mental character.

The word for medicine is the same as the word for the peyote cactus in many native North American tribes[17,18,19]. Other tribes have used the fungi of the species *Psilopsybe* (containing psilosybin) and the cactus called San Pedro (*Trichocereus pachanoi*), which like peyote contains mescaline as its major active substance. The liana called ayahuasca (*Banisteriopsis caapi*) has been used in South America, while other cultures like the ancient Egyptians used an LSD-like alkaloid derived from the Ergot of Rye, a plant disease caused by the fungus *Claviceps purpurea*[20]. All these drugs contain psychotropic (mind-expanding) drugs or hallucinogens, but with a very different profile from the recreational drugs in popular use among young people all over the world today[21] such as ecstasy, cocaine, and amphetamines, which have a strong CNS-stimulating effect in addition to a more modest, mind-expanding quality.

The purpose of the use of the mescaline-cacti among Native Americans is to bring the patient to a state of consciousness where he can realize how he makes himself ill by not living in accordance with the deep self[17], or in our interpretation, with the true human character and the purpose of life. This makes the fairly mysterious native medicine, often completely incomprehensible due to the use of massive symbolism, very difficult to understand. We are proud to say that the consciousness-based medicine we developed over the years does not use any kinds of drugs, as this has not been necessary because of highly efficient therapeutic techniques and strategies. The use of hallucinogenic drugs might be relevant in holistic medicine when patients only have very limited time and resources or are close to dying from cancer with metastasis to lung, liver, or other vital organs. Sometimes the patients ask if such drugs would be useful for them and the answer is that without a proper therapeutic setting to support the process of healing, it will probably be a waste of time and not beneficial. Sometimes the patients have been offered the drugs before in a therapeutic setting, as it often happens with travellers to the Amazon jungle being offered ayahuasca by the local medicine men (some talk about “ayahuasca tourism”). In such a case, it can be very difficult to help a patient further integrate the experience without using the drug again and only one such unintegrated experience of maximal intensity can be quite invalidating. If a hallucinogenic drug is to be used in the advanced or experimental holistic clinic, the most tested and traditionally most successful are without a doubt mescaline[17,18,19,20,21,22], psilosybine[20,21,22,23], LSD-25[20,21,22,24], and ayahuasca[25]. The effect of mescaline is the mildest, the easiest to control, and thus the less dangerous to use in a clinical setting, while psilosybine is stronger, with LDS-25 and ayahuasca traditionally regarded as the strongest hallucinogens.

It is very important to understand why previous attempts to use hallucinogenic drugs as medicine by western physicians have mostly failed and why the drug experience can be damaging[22]. It takes a high level of expertise to lead the patient into the holistic state of healing in general and presumably an even higher expertise to do this when the patient is on a psychotropic drug or any other drug affecting brain activity. The steps of "loving" the patient, winning trust, getting permission to give holding, and finally letting the patient surrender to the process of holistic healing is mandatory. A psychotropic drug can never do more than facilitate a process.

We visited the seven tribes of the Round Wally of Northern California in 2004 and could not find any medicine man that was able to bring his patients into the state of holistic healing using peyote or a similar substance. We met one medicine man who told us that the tradition of holistic healing using peyote was sadly lost in his tribe after the U.S. government moved the tribe from its original habitat almost 100 years ago. With the decay of the Native American culture, the tribes are not able to keep their old medical tradition alive and the wisdom of holistic medicine accumulated through the centuries is sadly lost.

To shed light on the close relationship between recovery of character (and purpose of life) and medicine let us take a closer look at the holistic process of healing.

## THE HOLISTIC PROCESS OF HEALING

New understanding of the holistic process of healing seems to cast light on these old procedures, which could give them a renaissance in the medical clinic. The life mission theory[1,2,3,4,5,6] states that everybody has a purpose of life and happiness comes from living out this purpose and succeeding in expressing the core talent in life. To do this, it is important to develop as a person into what is known as the natural condition, a condition where the person knows himself and uses all his efforts to achieve what is most important for him. The holistic process theory of healing[12,26,27,28] and the related quality of life theories[29,30,31] state that the return to the natural state of being is possible whenever the person gets the resources needed for existential healing. The resources needed are, according to the theory, in the dimensions of awareness, respect, care, acknowledgment, and acceptance with support and processing in the dimensions of feeling, understanding, and letting go of negative attitudes and beliefs.

The preconditions for the holistic healing to take place are trust and the intention of the healing taking place. Existential healing is not a local healing of any tissue, but a healing of the wholeness of the person, making him much more resourceful, loving, and knowledgeable of himself, his own needs, and wishes. In letting go of negative attitudes and beliefs, the person returns to a more responsible existential position and an improved quality of life. The philosophical change of the person's healing is often a change towards preferring difficult problems and challenges instead of avoiding difficulties in life[32,33,34,35, 36,37,38,39]. The person who becomes happier and more resourceful often also becomes more healthy, more talented, and more able to function[41,42].

## THE STRUCTURE OF HUMAN CHARACTER AND THE PURPOSE OF LIFE

The human character is seen to be a transformation of the abstract purpose of life[2] into the spirit, mind, and body of the human. The purpose of the character is to serve the purpose of life, giving every person the unique tools and means necessary for interindividual interaction: means for getting attention, obtaining contact, and influencing the other person according to the purpose of this person's life.

Fig. 1 shows a presentation of the relationship between purpose of life and the spiritual, mental, and physical character of the person and Fig. 2 shows the structure of the character and life purpose.

When we are conceiving the world, we need a structure, and when the purpose is medical scientific understanding of the world, the fractal ontologies seems to have major advantages to other structures. The fractal ontology can have bifurcations of any number with one as the trivial — there is one ray, undivided, and everything is understood as a simple product of this essential energy of the universe. Interestingly, this is the ontology of modern natural science where everything is seen as energy, whereas most of it is bound as matter making the atoms and molecules that constitutes everything and every cell of the body.

This is the position of biomedicine. The fractal ontology with the number two is the classical René Descartes (1596–1650) body-soul dualism[43] (see Fig. 3).

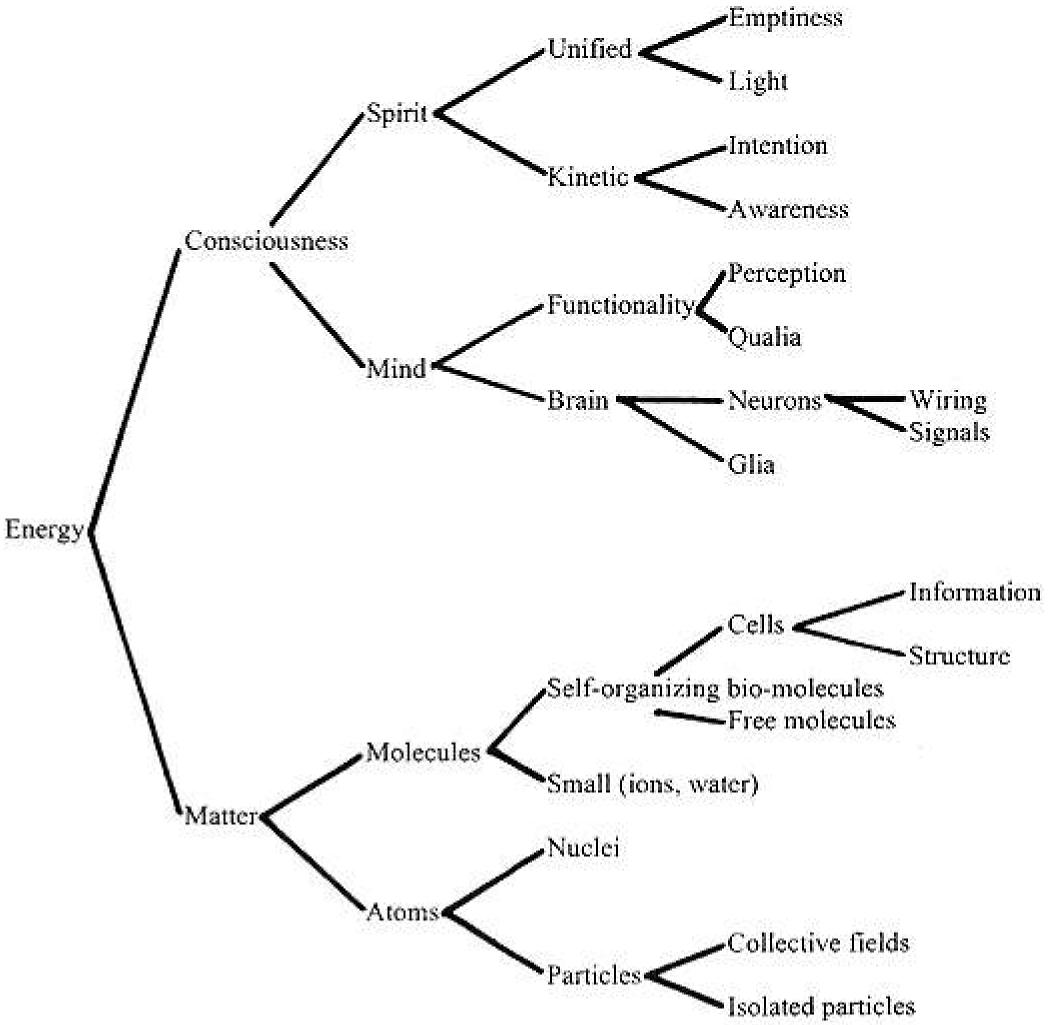

**FIGURE 3.** A fractal ontology with the bifurcation number 2. Except for the first lines, the concepts invited by this ontology are very much like the concept used by contemporary natural science. It seems that science, through time, developed towards acknowledging and integrating the concepts in the upper part of the scheme.

The interesting thing is that the more advanced the bifurcation is, the more continuous the description becomes. The two original halves of the world end up merging seamlessly. A three-ray ontology (see Fig. 4) is more complex, but the interesting thing is that the concepts invited by this structure seems to be more deep, complex, and more relevant to human consciousness.

## THE SEVEN-RAY THEORY OF CONSCIOUSNESS

Depending on the ontology and cultural setting, a wide range of words are used to describe the human character (see Table 1). Interestingly, some of the words are normally considered positive in civil life and negative in the martial arts, where the warrior must be powerful and destructive, while others are

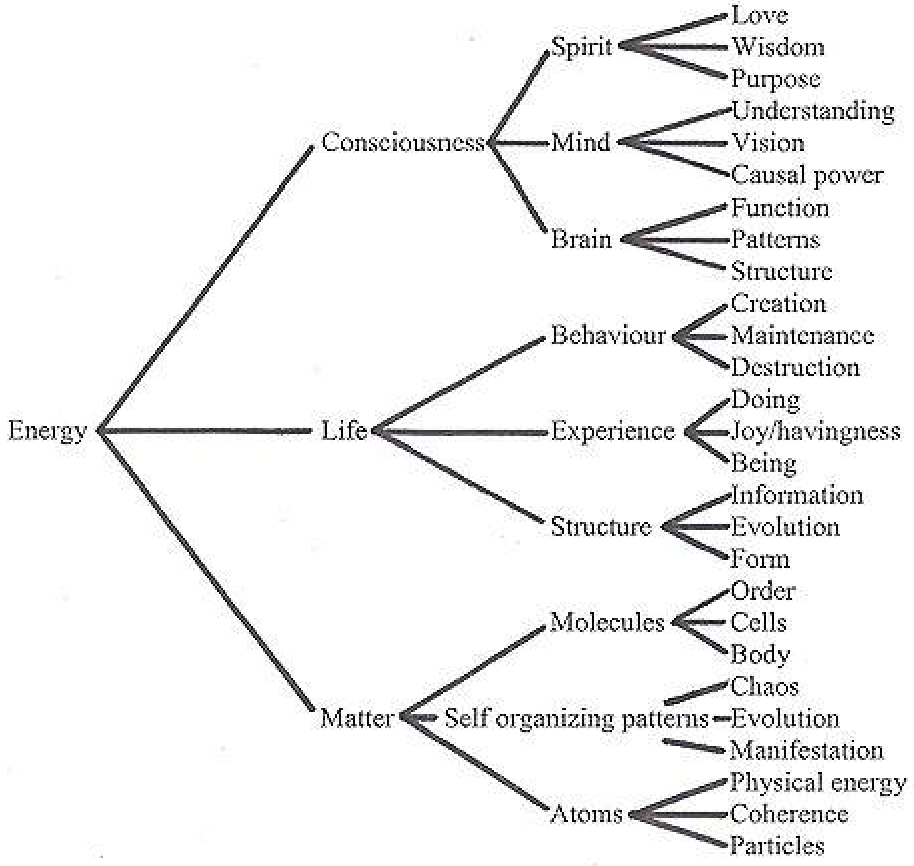

**FIGURE 4.** A fractal ontology with the bifurcation number 3. Please note that the concepts invited by a higher bifurcation number have more depth, complexity, and more appeal to the consciousness. They are more relevant to psychology and existential philosophy than to the natural sciences.

considered to be negative qualities in the civil life and positive in the martial arts. Interestingly, all of them can be seen as genuine aspects of human character and they are thus not evil, but mere tools for the soul. The intentions of the soul and the setting determine whether a dangerous or potentially negative streak is having positive or negative consequences. Often a negative quality will turn out to be a suppressed positive quality of the person's character and in the same way a positive character can turn out to be false and created by repression of a true "negative" character, serving the soul in its mission. We want a man, and a modern woman as well, to be kind towards us and also dangerous, powerful, and protective when this is necessary. The most convenient concepts for the use in the clinic with our patients describing their character — after having tried to use the language of astrology, Tarot (a deck of 78 picture cards that has been used for centuries to reveal hidden truths), I Ching (or "Book of Changes" is an ancient Chinese divination manual and book of wisdom), and others — seems to be the classical complex concepts from the old fractal theory of the energetic emanations of the universe[44] or "the seven[four, five, six?] rays of energy"[45] known in a number of versions and originally from both ancient India and Egypt. Interestingly, the origin of the theory seems to be the cultures' medical systems and esoteric healing has sought to use them up to this day[46,47].

"The seven rays" are the answer to the eternal question: what exists? In natural science, we often give the answer "physical matter only", which obviously is a kind of one-ray theory. Descartes[43] said that what existed was body and mind, matter and consciousness, which is a two-ray theory. In our study group

"Matter, Life, and Consciousness", we have worked for years on a three-ray theory to show that these three aspects of the world comprise what is needed for a full scientific understanding of reality (see Fig. 4). The seven-ray theory is highly complex compared to this, as every one of the seven rays, or basic concepts, consists of a different mixture of matter and consciousness, in a spectrum from very little to very much; the following "seven elements" symbolize the different "mixtures" (with symbolic relation between consciousness/matter):

| | |
|---|---|
| 7) consciousness | 1000/1 |
| 6) spirit | 100/1 |
| 5) ether | 10/1 |
| 4) fire | 1/1 |
| 3) air | 1/10 |
| 2) fluent | 1/100 |
| 1) solid | 1/1000 |

Here, the classical four alchemic elements are the basis symbols, with the three old esoteric "elements" on the top. Needless to say, these elements are not to be taken literally as existent. We are talking about the qualities of the universe when seen through the "prism of life" — in an analogy to the way light is seen as seven rays of different colors and frequencies in a glass prism. So we do not take this to be a physical ontology, but only a consciousness-related, man-related, subjective ontology. But this gives us exactly what we need to describe the character of man — a language to work with, making the most subtle nature of man possible to operationalize in the holistic clinic.

Spiritual character is the most subtle, closely connected to the most abstract aspect of the wholeness or soul (the purpose of life). Gurdjieff thought that our spiritual character is one of nine aspects of Good that we mirror in our existence[13] and this idea gave rise to his enneagram systems, very popular with business leaders worldwide today. One aspect of Good is truth, another love, a third perfection, a fourth will, a fifth the laws of the universe, a sixth the divine creative source, the seventh omniscience and wealth, the eighth trust, and the ninth the divine plan according to which the whole universe unfolds.

Mental character is our preferred way to deal with reality. Our theory is that early in life we are able to use all our mental talents, but as the pressure builds up on our existence, we must choose to develop and perfect one of these mental strategies to be able to survive. The mental characters are traditionally described in connection with the different levels of the body in centers (chakras) carrying their own intelligence. It seems that these levels of the body actually reflect this activity as a lack of presence in these areas of the body and are often connected with a lack of the parallel talent. We believe that useful tools should be developed to assist our patients to improve their quality of life, health, and ability to function.

The scientific basis for the seven-ray theory of consciousness is an understanding of the patterns of the working brain coming from theory of fractals, chaos, and complexity[35,36]. However, there is yet no theory to explain how the brain can stabilize in the different "fractal modes" corresponding to the series of poly-ray ontologies giving the different interpretations of the world, and thus the subjective universe of the human characters. The intersubjective quality of character and consciousness is not well explained by current neurological and brain theory.

## THE SEVEN RAYS IN DIFFERENT CULTURES

The most popular of the poly-ray ontologies seems to be the seven-ray ontology[45]. The Hindus speak of the "Seven Praj patis" (Lords of Creation); the Zoroastrians of the "Seven Amesha Spentas" (Immortal Holy Ones); in Egypt they were the "Seven Mystery Gods"; in the Christian Bible, we meet the seven spirits[48]: "Before the throne[of God], seven lamps were blazing. These are the seven spirits of God"; and in the Theosophical concept, we find the term "Seven Planetary Chain Logoi".

The seven rays are also called the seven spirits and they are responsive to the seven sacred planets and expressions of divine life on Earth. In the esoteric psychology, Alice A. Bailey talks about the Seven Primeval Rays, or seven planets: "There are seven chief planets, the spheres of the in dwelling seven Spirits [49]."

Different cultures seem to have used different numbered fractal ontologies: the medieval alchemic researchers used the number 4[50], using the concept of the four elements; the old Chinese philosophy used the number 5[51]; the Upanishads and old Indian philosophy used the number 7[49]; the old Egyptian mysticism used the number 9[53]; and the Kabbalah or Jewish mysticism used the number 10. In the Jewish Kabbalah, there are ten rays called the "Ten Sephiroth"[54,55] and this system is often said to be more complex and thus more difficult to learn and understand than many of the other esoteric systems. Strangely, the higher number the more complex and incomprehensible — the more mystic and intuitive — is the philosophy.

It seems that the lower the number, the more materialistic the ontology and the higher the number the more spiritual. A high-numbered ontology seems to invite concepts and structures close to the inner nature of our consciousness. We chose a medium-range complex ontology for our work, a seven-ray ontology, which makes a good and not too complex theory for human consciousness.

## THE DISCRIPTION OF THE HUMAN CHARACTER

As these concepts are used here to describe fundamental qualities of consciousness, it is important to understand the rays symbolically. The seven rays are hardly the ontological description anyone would use in modern natural science, but look how nicely it gives us the fundamental concepts of human character (see Table 2).

**TABLE 2**
**The Major Spiritual, Mental, and Physical Characters**

| **Ray of Energy** | **→** | **7** | **6** | **5** |
|---|---|---|---|---|
| **↓** | | **Spirit [13]** | **Mind** | **Body [56]** |
| 7 | Consciousness | Truth, perfection | Understanding | Intelligence |
| 6 | Spirit | Devine plan, do | Vision | Kinetics/eccentric |
| 5 | Ether | Will | Power/essence | Electric/danger |
| 4 | Fire | Love | Heart* | Charm/warm |
| 3 | Air | Trust, have | Relations | Humor/light |
| 2 | Fluent | Omniscience, be | Alignment | Symmetry/beauty |
| 1 | Solid | Source, law | Integrity | Solid/physical |

*Can be further divided[57]: inspiration (ray 6), insight (ray 2), integration (ray 1).

The seven subtypes of the 3 × 7 key characters are derived from the iterative permutation with the seven rays; seven subtypes of the physical types are listed in Table 3. The words chosen here are strongly inspired by Henry Kimsey-House[56,58] and are only a few of the many relevant words for the human physical character; every character has seven subtypes and so forth.

We believe that the seven types of love, relationships, and sexuality similarly can be mapped and described (with words such as lust, ecstasy, euphoria, passion, romance, coherence, and oneness), but we have not yet made this scheme a useful tool in the clinic. The spiritual and mental subtypes (described with words, i.e., for the divine plan: time and space, value, leadership, responsibility, resources, success and transcendence; and for the mental hearth: grace, acceptance, respect, affinity, honor, awareness, wholeness) are so integrated in our daily language that the mapping seems fairly trivial.

**TABLE 3**
**The Subtypes of the Human Physical Character***

| RAY | 1 Solid (Sextype) [56] | 2 Fluent (Symmetric, Beauty) [56] | 3 Air (Light, Humor) [56] | 4 Charm (Warm) [56] | 5 Electric (Danger) [56] | 6 Kinetic (Eccentric) [56] | 7 Intelligence |
|---|---|---|---|---|---|---|---|
| 7 | Glittering (diamond) | Royal | Clown | Suave | Overwhelming | Arrogant | Wise |
| 6 | Exotic | Aloof | Ironic | Easy going | Psycho | Unpredictable | Brain |
| 5 | Ethereal | Romantic | Zinger | Elegant | Threatening | Sly, wacky | Wit |
| 4 | Shy | Magical | Cynical | Graceful | Killer | Subtle | Authority |
| 3 | Cuddle | Playful | Cocky | Hip | Scary | Spacey | Experimental |
| 2 | Sexy/ steamy | Innocent | Neurotic | Slick | Smoldering | Bold | Enigmatic |
| 1 | Sultry | Natural | Nerd | Earthy | Methodical | Awkward | Sophisticated |

*The words are only examples.

## IDENTIFYING HUMAN CHARACTER IN THE CLINIC

It takes quite a while to be so accustomed to the categories of spirit, mind, and body that the qualities are discriminated without problems by the holistic physician. Actually, it is often quite easy to recognize them when the patient acknowledges them in him- or herself. But when the characteristics are repressed by the patient modifying his own character by intending the opposite and thus annihilating the original character as a strategy for coping and survival, it often turns complicated. What is often left of the human character is only a symptom of physical, mental, or spiritual disease:

| True Character (Physical Type, Five Subtypes) | False Character (Many Variations) | Symptom |
|---|---|---|
| Arrogant | Kind | Weak |
| Intelligent | Stupid | Dull |
| Sexy | Cheep | Ugly |
| Sultry | Nursing | Gone, disappeared |
| Steamy | Clammy | Asexual |
| Killer | Nice | Dead |
| Scary | Unimportant | Depressed |
| Smoldering | Hot | Annoying |
| Methodical | Messy | Weird |

So here is an example: you see a seemingly, very nice patient who is giving you the feeling that he is completely dead inside. You realize that he hides something and when you dig into this strange, messedup area of the patient's personality and existence, it turns our that he is a real "killer" (with a strong personality like the great actor Jack Nicholson), but this is repressed. The "killer" character is a beautiful, powerful personal quality when you take it as strength or as something that helps you fulfill your purpose of life. It is very important not to confuse character with intent, as people often do. Early in life, this patient had parents who could not see the positive and valuable in their son being "a killer", so they made him repress this vital characteristic and changed him to be "nice" in order to fit into the family and receive the love and acceptance he badly needed for his survival. And ever since, he has been more or less dead and not able to unfold his fine character, his finest social tool, and thus never had the impact on others necessary for success.

## RECOVERING HUMAN CHARACTER IN THE HOLISTIC CLINIC

To put a word on the character of the patient is a delicate matter. If the physician uses the wrong word or mistakes a suppressed character for being the real, he can do serious harm to his client. The work with character must therefore be done in a dialog, with the client having the last word on the matter. The physician must challenge the patient when he or she feels that something is not right or completely clear and must be brutally honest, but as none of us are pure, we can and will make mistakes and room must be made for that, continuously stating our own limitations to the patient.

On the couch, the patient can be helped to concentrate on her self, spirit, mind, and body, contemplating and reconsidering her deep nature and character. A fine thing to do is to make the patient find out on his or her own, as a home exercise, what the true character is — spiritually, mentally, and physically — and discuss it afterwards in the clinic, which often bring about huge revelations.

The following case story is about a young woman who had completely lost her touch and connections with her deep self. She had turned into something very different and much less appealing than her true character. The case story illustrates how deeply our life, body, and whole physiology is influenced by the suppression of our character and how efficient the existential healing is. The healing will be on the physical, mental, and spiritual level, when the patient finally recovers her character.

The theories taught to the patient are the theories of existence in the life mission theory series[1,2,3,4,5,6]. The techniques are described in the book on consciousness-based medicine[59] and a series of papers on clinical holistic medicine[60,61,62,63,64,65,66,67,68]. In the following case, the patient is treated in accordance with the holistic process theory of healing[27] with a focus on recovering the character and purpose of life. Only after 11 holistic sessions was she able to see who she really is and in this awakening, she recovered her character and her health.

## CASE STUDY

**Female, 36 years, menopause too soon, existential problems:** Holistic session 1: Menstrual problems for the past 2 years and the gynecologist believed it to be a hormonal disorder. Gynecologist found a large cyst at left ovary, which had disappeared a year later. Menstruation always 21+6–7, but last year only 20 days and over the past year from 19 days to 18–17 days. Menstruation absent for the past 5 weeks. Gynecologist made the diagnosis/ early menopause/ FSH at 72, estrogen: immeasurable values. Caesarean section in 1994, no sequelae. Socially: university degree, social education. Work as a consultant and project manager at IT company; usually manager for about 10 employees, customer care, etc. She also does sales. Hard working and called "The Corporate Bitch", "Stalin", etc., because she is tough, efficient, and controlling. "I never thought of myself as very feminine," patient says. Suspicion: Early decision: "I am a boy." Partner is big and strong, but very sweet and soft — feminine? Objectively: Extremely well functioning and fast. Appears feminine in a masculine way, very sweet and thoughtful, cries when we talk about how she really feels. “I don’t feel very well right now,” she says, and “it’s a fight [to live].” She adds: “It feels almost liberating to have reached menopause.” She appears invulnerable; I feel that she in her natural state would be far more vulnerable. /Masculinization/ We talked about alienation and the purpose of life theory, including subtalents. She does not seem very introspective. EXERCISE: be vulnerable and sensitive. Every time you feel an emotion, stop and feel it. Every time you get hurt, stop and feel hurt instead of acting on your impulse. New appointment in 1 week

Holistic session 2: Has been doing a lot of thinking since the last session. Considers quitting her job. Realizes that she has pushed herself way too hard. Her period returned last Friday. It felt like real menstruation with lots of cramps. Realized that she spends perhaps 95% of her life energy at work. Told her colleagues that she has doubts about her future job function and she was offered partnership. Plans to report sick for 14 days due to stress. We talk about the inner garbage bin and about developing oneself to be oneself. Wants to set up a therapy contract and make a plan of development and treatment. We will do that next time.

Holistic session 3: We talk about the dimensions of talent, power and gender — about re-establishing the natural condition[4]. “Men are wimps” — an example of negative essence regarding gender. We plan the phases of the treatment: First trust, then processing. It will take approx. 6 months. Rosen method therapy is an option, acupuncture is another. Is it possible to work with her body? New appointment in 14 days.

Holistic session 4: Bleeding occurred too early after last period (+15/16 days) and too short (3 days); period was normal, but then disappeared. Partner has suffered from erysipelas for 14 days, is rather ill. Patient does not see any connection. We work on the couch with closeness and intimacy; she says she likes to be dominated sexually. We talk about her “prostitute” quality — that she acts instrumentally and not lovingly with other people; “you” has become “it”. The patient prefers to use the language of the chakras; we talk about how she is present in her root chackra[first ray], solar plexus[third ray], throat-[fifth ray], and perhaps crown chakras[seventh ray], less in the third eye [sixth ray] and only a little bit in the second chakra (hara centre) [second ray]. I hold my hands over her uterus, it is as if she is not present here. She cries a little, when we talk about her having to become human and have a life. NOTE: This patient will not remove her boots when she is lying on the couch; she wears small underwear and boots, which gives her a necessary sense of feeling safe. During the session she processes a lot of embarrassment and shame that she will not acknowledge. EXERCISE: When you feel something, stay in the emotion and make it as strong as you can. WARNING: Emotionally, you may become rather unstable. New appointment in 14 days.

Holistic session 5: Went to see her gynecologist and FSH has stabilized from 71 to 2.3, so she is no longer in menopause. He wondered whether the blood specimens had been swapped. I ask for a copy of her gynecologist report. She will consider retrieving it for me. We discuss her further development; she can continue here for life purpose processing. We discuss a strategy: when she is present in mind and body she can conquer feelings in her heart and the door to her soul will probably open. During this session we happen to hurt each other and we have a good talk about vulnerability and the purpose of being alive, as it is possible to get hurt.

Holistic session 6: She feels better, but unstable. She feels very sad and sees herself as being destructive when considering getting a new boyfriend; she feels that her old relationship no longer gives her what she needs. We talk about the old having to be torn down before the new can be built. She had a rash, which she felt ashamed of, that is why she wanted to keep on her boots on the couch. On the couch, we work with the fact that "she often has mental blocks". We discuss the two layers in her — a false and superfluous one, and a true and deep one; when I reach the limit between the two layers, she twists her existence and falls into a mental block. Her body and stomach feel like overcooked chicken meat. We discuss why: because she is not well connected inside. We discuss orgasmic potency — she could become more sexual. I think she looks lovely today and that everything is progressing very nicely for her.

Holistic session 7: Went to her gynecologist, she has to bring me the report. The numbers are irregular, her period is still unstable, the mucous membrane is not quite thick enough. She is ovulating; blood work confirms this. She is a bit hyper. She still works way too much — 50 hours per week — doing intellectual concept development. She is 80% in her head and 20% in her stomach and her uterus cannot function under those circumstances; that is how I see it. She does not seem to really love her new boyfriend; she likes someone else, but she does not really love him either. "It's been years since I really loved a man," she says. We discuss her values, and I make her say: "Sex is not important. Love is not important. Power is important (money, control, speed, etc)." She started attending a course in "Corporate NLP"; I advise her to stop. She does not need more mental material. On the couch, we work with acceptation by touching her abdomen. We continue down to her pubis. Her uterus feels "cold" and rejected. I am slightly frustrated by the fact that her intuition regarding her needs and her situation is so weak. EXERCISE: What is your purpose of life? Do (?): Is love important to you? Describe on one page, letter size, what love is to you. Do (?): Read "The Prophet" by Kahlil Gibran. New appointment in 14 days. We need to consider a gynecological examination.

Holistic session 8: Patient brings her medical report. Her gynecologist writes before start of my treatment: "Gonadotrope hormones show manifest climacteric values; it indeed looks like climacterium praecox"; and now: "Apparently reversed to spontaneous cycle." She tells me she has become fond of another man and that she no longer feels like being with her boyfriend sexually. She is not leaving him, though, and will not tell him the truth because the relationship gives her the following advantages: safety, he is nice to be with and patient with her — a friend! She gets turned on by someone else. She tells me that she was sexually active with a girlfriend at 7 years and that her father discovered them and took a picture "to remember this day of shame". EXERCISE: What is your purpose of life? She did her homework. She brings lots of love and feelings. Is this what life is all about? "I can't even sense that I have a purpose in life!" I humbly ask her to search one more time. EXERCISE: Is love important to you? Yes; "Love is more important than power." Describe on one page, letter size, what love is to you.

Holistic session 9: She is doing great. She broke up with her boyfriend, found a new and very nice 35-year-old man who wants to settle down and she has become much happier and lovelier. "Stupid and lovely," I say. She does not like that. But she really has become so. Her words are far apart, and she has begun to pronounce them as a "stupid blonde". On the couch we work with her stomach and the fear she needs to feel. EXERCISE: Be slow, stupid, lovely, and afraid. Breathe all the way down your stomach, all the way to the bottom of your pelvis and the opening of your vagina. EXERCISE: Try to be receptively sexual; feel without trying too hard; the orgasm should occur in the center of the pelvis.

Holistic session 10: Has trouble standing her job. Went to her gynecologist again for more blood work because she had irregular periods after meeting her new boyfriend. Silly girl. “What do you want to do with that?” I ask. She just acts silly. We have talked about her only working 30 hours per week because otherwise she cannot live her purpose fully. Her purpose has to do with being very feminine and very sweet. “I am sweet and lovely,” I ask her to say, but she says: “I am evil and manipulating.” She says, “I am sweet and lovely” 100 times and gradually understand the meaning; she cries a little, but still wants to be the corporate bitch. That is what the job takes. She is a project manager. EXERCISE: Spiritually: Management should come from the sweet and lovely aspect of your personality. Sweet like honey, lovely as the most beautiful music. Bodily: Determined, efficient, powerful bitch. Music. Mentally: 3. Eye — sharp and confronting like hell.

Holistic session 11: Finished at the gynecologist, she was measured again (hormone number is at 31), this means she has been ovulating (the min. limit is 20). Period is stable at +21 days. She is confused and sad and needs a plan. She is annoyed with her new boyfriend, who is extremely boring. The theory of character is discussed and we reach the following: Purpose of life: I love life (1$^{st}$ try). Spiritual character: Mastering the laws of the world (with “will” + “source”). Mental character: Alignment, connection. Physical character: Water/Beauty (symmetry, balance); charming, romantic, ivory, bold. EXERCISE: Live your character fully: I love life — everything about it!!!! Use your character to do it! EXERCISE: Read the book by Maitri: *The Spiritual Dimension of the Enneagram*[13].

## DISCUSSION

Character is not a term commonly used in science and social science literature. The reason for using this term is the unique intersubjective aspect of the human being known from the arts, theater, and the grand epic literature of the world cultures. It seems thus that character is an important aspect of life in need of a scientific exploration. The presented theory integrates a vast number of prescientific and psychoanalytically inspired theories, where the idea of repression of the character into the subconscious is central in all the life mission theories[1,2,3,4,5,6] and the theory of holistic healing[27,28,60,61,62,63, 64,65,66]. The disadvantage of this “Freudian” flavor is a feeling of mystery, as it really is difficult to understand the mechanistic and neurological principles according to which gestalts can be buried in our unconsciousness. But however mysterious from a natural science point of view, the unconscious (as well as memory and consciousness) is a subjective as well as clinical reality.

Our attempt in this paper has been to map the aspects of the human character relevant for the holistic clinic. As this is a new area of research, we do not conceive the presented tables as final; on the contrary, we expect to accumulate experience and refine our scheme and our clinical tools in the future. From our clinical experience with several hundred patients working on recovering their character, we find that this work has excellent therapeutic value. In many cases, it might actually be the most efficient way to rehabilitation and general recovery because our purpose of life is so intimately connected to our character, that when we recover our character, it is almost inevitable that we will understand our true nature and personal mission of life in the end.

The problem of helping the patient to realize his or her character is important and not elegantly solved. In the clinic, the identification of the character is done by agreement between the therapist and the client, but often the acceptance of one character leads to the discovery of a new layer, hidden behind the first, so the process is often repetitive and lengthy. It is very important to underline that character can be mentally projected, so the therapist must be in intensive dialog with his patient and only the full healing of the patient in all aspects of health and quality of life is the final proof of a job well done. We also believe that this is connected to the revelation of the purpose of life (the life mission). Holistic medicine using the theory of character is thus only an extension of existential therapy.

It is very much like a simple riddle: This car has huge, broad back tires, small front tires, a very strong motor, and a little house where you can hide from bad whether — what kind of car is it? Oh, that must be a tractor meant for agriculture. When you know the characteristics, you can easily get the idea of its purpose also. On the other hand, if you cannot see the vehicle, it might be fairly difficult to guess what it is meant for. Therefore, knowing the meaning of life, living our purpose, and using all our gifts and talents are what we are meant to do; this is what makes us truly sound and happy.

The recovery of purpose of life and human character has not been a subject of the western medicine only. Interestingly, there are strong parallels to the medicine of the Native Americans[69], the African Sangomas[70], the Australian Aboriginal healers[71], and the old Nordic medicine[72]. We believe that we now are at the fundamental core of consciousness-based medicine, since recovery of purpose of life and human character has been practiced as medicine universally.

It is very important not to ignore the efficiency of manual medicine and biomedicine when working with consciousness-oriented medicine. Every patient must be examined and diagnosed according to best medical practice, so all treatment alternatives are found and presented to the patient, so the patient together with the physician can find the best and most appropriate treatment plan. It is crucial that a consciousness-oriented treatment not become a hindrance to life-saving surgery or other well-documented procedures, which with a high certainty can help the patient. On the other hand, a holistic treatment will ensure the focus of the patient to learn from the disease and improve quality of life, so the art of the multiparadigmatic physician[62] is really to balance the different methods in the best interest of the patient. The physician must follow his instinct with every patient and the patient must be fully informed to give his or her consent to the proposed combination of examination and treatments. As holistic medicine often is administered to chronic patients without much hope for cure by biomedicine that has failed to cure them already, the biomedically oriented physicians must have some understanding for the patients giving their trust and hope in the holistic treatment, even if this can mean that the patients are not living as long as they could when using all the facilities of a modern biomedical hospital unit. Quality of life is not just about survival, but also about fighting for life in the personal way one finds it right to fight it.

This brings us to the necessary remarks on ethics. Holistic medicine needs more than manual and biomedicine ethics to succeed. We believe that only when the physician is working with his heart, using the fourth ray and giving his patient his full support and unselfish service, will holistic medicine work. When the holistic physician can do this, he can work with all the parts and all the rays of the patients, all aspects of the patient’s life, the consciousness, the physicality of the body, the sexuality, the organs of the body, the most intimate emotions, the deepest confidentiality, the love, the hope, and the belief of the patient.

If the physician does not come solely from his heart, with the intention of curing as the only intention regarding this patient, the patients will feel insecure, lack trust, maybe even feel abused or let down, and the holistic treatment will most likely fail to bring the patient into the state of holistic healing and cure. Loving the patient is a prerequisite, an absolute condition, for making holistic medicine work.

An interesting aspect of the theory of character is that it seems to explain the practice of homeopathy. The patient’s consciousness is guided towards the fundamental repression of the physical, mental, and spiritual character. The homeopathic remedies, which we believe to be sheer placebo preparations, have the important aspect of keeping the patient’s consciousness focused on the symptoms and the repressed character, helping the patient to feel deeply into the problem, understand the dynamic of it on an emotional level, and finally let go of the negative beliefs creating the disease.

Finally, let us discuss some of the many pitfalls of holistic medicine, especially when taking it into the field of intersubjectivity and human character. The most problematic side of doing this is that we lose the scientific clarity that we have worked so hard to obtain in our science. In a way, we tried to clear everything up just by ending in a new mystery. One other difficulty is that using theories of this level of complexity makes understanding, practicing, and teaching holistic medicine difficult and complicated. The risks of misjudgments and projections become substantial with the theory of character. Another problem is that using such a theory can give the patient a feeling of the physician possessing "expert knowledge", which creates a distance and a hindrance in holistic medicine, where intimacy and trust is a must. Finally, such theories can invite therapists with unclear minds to justify their lack of insight and understanding, refereeing to the inherent mysterious nature and qualities of consciousness. All these pitfalls must be avoided at all cost for holistic medicine to be truly helpful to the patient.

## CONCLUSION

We have presented a theory of the human character and the purpose of life and explained the relevance to existential healing. We have suggested a scheme of the major human characters for use in the clinic, but the qualities listed are not physical or objective qualities of man, instead it is quite the opposite: highly subjective qualities directly connected to the consciousness and purpose of life of the person. It seems that our purpose of life can only be realized if we acknowledge our own spiritual, mental, and physical character; recovering human character seems, therefore, to be of major importance in clinical holistic medicine.

We believe that quality of life, physical and mental health, and general ability to function can be radically increased when the patients recover their character and understand their purpose of life by living accordingly. Now we need research in this field to document the efficiency on patients with a number of different diseases, not helped much by other kinds of treatment, biomedical, manual, and alternative.

We believe the concept of recovery of character to be one of the important aspects of Hippocrates' medicine, reused by Hahnemann in his homeopathy. We hope when developed, this upgrade of holistic medicine can be helpful to support the immune system of HIV patients[73] and to help with inducing spontaneous remissions of cancer[73,74] and other diseases[60,61,62,63,64,65,66,67,68, 75,76,77,78,79].

We strongly encourage governments and foundations to support this research in holistic medicine with the necessary funding. We also would like to invite the medical community to a long-term cooperation, solving the huge problems of holistic medicine[26].

## ACKNOWLEDGMENTS

We wish to thank Henry and Karen Kimsey-House, as important inspiration to this work came from their teachings on Henry Kimsey-House's "I Am Type" system on the physical character at Co-Active Space leadership program. Our research in quality of life has been was supported by grants from the 1991 Pharmacy Foundation, as well as by supplementary grants from Goodwill-fonden, the JL-Foundation, E. Danielsen and Wife's Foundation, Emmerick Meyer's Trust, the Frimodt-Heineken Foundation, the Hede Nielsen Family Foundation, Petrus Andersens Fond, Wholesaler C.P. Frederiksens Study Trust, Else & Mogens Wedell-Wedellsborg's Foundation and IMK Almene Fond. The quality of life research was approved by the Copenhagen Scientific Ethical Committee under number (KF) V.100.2123/91.

## BIOSKETCHES

**Søren Ventegodt, MD,** is the Director of the Quality of Life Research Center in Copenhagen, Denmark. He is also responsible for a Research Clinic for Holistic Medicine in Copenhagen and is a popular speaker throughout Scandinavia. He has published numerous scientific or popular articles and a number of books on holistic medicine, quality of life, and quality of working life. His most important scientific contributions are the comprehensive SEQOL questionnaire, the very short QoL5 questionnaire, the integrated QOL theory, the holistic process theory of healing, the life mission theory, and the Danish Quality of Life Research Survey, 1991–94 in cooperation with the University Hospital of Copenhagen and the late pediatric professor Bengt Zachau-Christiansen. E-mail: ventegodt@livskvalitet.org. Website: http://www.livskvalitet.org

**Maximilian Kromann, Cand.Phil**. His main field is existential philosophy and he has read more than 1000 specialist books regarding this subject. His most important scientific contributions are the understanding of the nature of man and the preconditions for a successful self-expression. He is also the director of the Quality of Life Bookstore in Copenhagen, Denmark, where he is coaching patients and customers in selecting literature that can develop their personal philosophy of life, and he is a popular speaker on his topic.

**Niels Jørgen Andersen, MSc,** Professor, Department of Innovation and Economic Organization, Norwegian School of Management. This department conducts research and provides teaching in central topics related to innovation, business development, management of global companies, business history, and economic organization. Research activities within the department are related to four core subjects within the discipline: business history, cooperative organizations, business development and entrepreneurship, and finally studies of industries with a special focus on the electricity industry. He is also the dynamic chairman of the nonprofit organization Stiftelsen Holistisk Medisin Scandinavia, that aims to support the scientific development, research, and documentation of complementary and holistic medicine in Scandinavia. E-mail: niels.j.andersen@bi.no. Website: www.bi.no/users/fgl93013/

**Joav Merrick, MD, DMSc,** is Professor of Child Health and Human Development affiliated with the Zusman Child Development Center, Division of Pediatrics and Community Health at the Ben Gurion University, Beer-Sheva, Israel; the Medical Director of the Division for Mental Retardation, Ministry of Social Affairs, Jerusalem; and the Founder and Director of the National Institute of Child Health and Human Development. He has numerous publications in the field of child and human development, rehabilitation, intellectual disability, disability, health, welfare, abuse, advocacy, quality of life, and prevention. Dr. Merrick received the Peter Sabroe Child Award for outstanding work on behalf of Danish Children in 1985 and the International LEGO-Prize ("The Children's Nobel Prize") for an extraordinary contribution towards improvement in child welfare and well being in 1987. E-mail: jmerrick@internet-zahav.net. Website: www.nichd-israel.com
